# A Retrospective Analysis of Clinical Outcome and Predictive Factors for Responders with Knee Osteoarthritis to a Single Injection of Leukocyte-Poor Platelet-Rich Plasma

**DOI:** 10.3390/jcm10215121

**Published:** 2021-10-31

**Authors:** Naoya Kikuchi, Tomokazu Yoshioka, Norihito Arai, Hisashi Sugaya, Kojiro Hyodo, Yu Taniguchi, Kosuke Okuno, Akihiro Kanamori, Masashi Yamazaki

**Affiliations:** 1 Department of Orthopedic Surgery, Faculty of Medicine, University of Tsukuba, 1-1-1 Tennodai, Tsukuba 305-8575, Ibaraki, Japan; nhskikuchi@md.tsukuba.ac.jp (N.K.); narai@tsukuba-seikei.jp (N.A.); pjxgr965@tsukuba-seikei.jp (K.H.); okun0nuko@tsukuba-seikei.jp (K.O.); kanamori@md.tsukuba.ac.jp (A.K.); masashiy@md.tsukuba.ac.jp (M.Y.); 2 Division of Regenerative Medicine for Musculoskeletal System, Faculty of Medicine, University of Tsukuba, 1-1-1 Tennodai, Tsukuba 305-8575, Ibaraki, Japan; 3 Department of Health, Faculty of Health Sciences, Tsukuba University of Technology, 4-3-15 Amakubo, Tsukuba 305-0005, Ibaraki, Japan; h.sugaya@k.tsukuba-tech.ac.jp; 4 Department of Orthopaedic Surgery, Ichihara Hospital, 3681 Ozone, Tsukuba 300-3295, Ibaraki, Japan; cindy.forever911@tsukuba-seikei.jp

**Keywords:** plasma rich in growth factor, platelet-rich plasma, knee osteoarthritis

## Abstract

Although various platelet-rich plasma (PRP) kits are commercially available, the efficacy of these kits for knee osteoarthritis (KOA) has not been fully investigated. This study aimed to investigate the short-term results of leukocyte-poor PRP (LP-PRP) and the factors that contribute to its efficacy. We retrospectively reviewed 124 patients with KOA who were treated with LP-PRP. White blood cell (WBC) and platelet counts in the whole blood and the LP-PRP were measured. KOA severity was assessed using radiography. Clinical evaluation was performed both prior to injection and after an average of 3.3 weeks after the injection using the Japanese Knee Osteoarthritis Measure (JKOM). Responders were defined based on the JKOM. The contributing factors for responders were examined using a multivariate logistic analysis. The responder rate was 58.1% and the contributing factors for responders were a higher visual analog scale score before injection, WBC count in whole blood, and platelet concentration ratio of LP-PRP. The LP-PRP improved the clinical scores in the short term. Certain patient characteristics before injection and the concentration ratio of LP-PRP may be predictors of its efficacy; these may provide clues for elucidating which components of LP-PRP act on KOA pathologies.

## 1. Introduction

Knee osteoarthritis (KOA) is considered “a whole-organ disease of the joint” [1] and causes joint pain and loss of function [2]. The prevalence of KOA increases with age [3]. Although nonsteroidal anti-inflammatory drugs, hyaluronic acid (HA), and steroids are widely used for the conservative treatment of KOA, platelet-rich plasma (PRP) has been attracting attention as a new treatment modality.

PRP therapy involves a complex process in which growth factors found within concentrated platelet alpha granules, cell adhesion molecules, and glycoproteins in the plasma act on tissues while maintaining a physiological balance within the body [4,5]. The efficacy of PRP for KOA has been demonstrated in systematic reviews and meta-analyses [6,7]. However, there are multiple PRP-purification systems [8,9], and the platelet concentration, degree of leukocyte enrichment, and concentration of the growth factors in PRP vary greatly depending on the preparation method [10,11]. Chala et al. [12] noted that approximately 90% of the previous studies did not report a clear description of their preparation protocol. Thus, it is desirable to understand the characteristics of each PRP system and apply them to clinical practice.

Plasma rich in growth factor (PRGF) is a leukocyte-poor PRP (LP-PRP) that was developed in the dental field in the late 1990s [13]. It has since been used in the orthopedic field and has been applied to the treatment of tendons and ligaments [14,15]. The clinical results of PRGF for KOA were reported by Sánchez et al. [16] in 2008. Subsequently, randomized controlled trials have shown a superior efficacy of PRGF over that of HA [17,18]. A systematic review has also reported the efficacy and safety [19]. In addition, PRGF injection for KOA could delay total knee arthroplasty [20]. Immediate response to therapy is clinically important, and understanding the factors associated with response to therapy is indeed a relevant clinical need.

The purpose of this study was to assess the clinical outcomes of LP-PRP for KOA at a mean of 3 weeks after injection for identifying the percentage of patients who met the modified Outcome Measures in Rheumatology and Osteoarthritis Research Society International consensus responder criteria (OMERACT-OARSI) [21], and to investigate predictive factors for responders.

## 2. Materials and Methods

### 2.1. Participant Recruitment

We retrospectively reviewed patients with KOA who were treated consecutively with LP-PRP injections from October 2018 to December 2020. The indication for LP-PRP therapy was being older than 20 years old with symptomatic KOA (diagnosed by clinical and imaging investigations). The inclusion criteria were knee pain and functional impairment resistant to known conservative therapy and the presence of KOA, as demonstrated by radiography. The exclusion criteria for patients were hemoglobin levels less than 9.0 g/dL, congealing fibrinogenolysis system disorders, and systemic inflammatory diseases. Written informed consent was obtained from all patients prior to LP-PRP therapy and all procedures were performed in accordance with the 1964 Helsinki Declaration and its later amendments.

### 2.2. LP-PRP Preparation and Injection

Thirty-six milliliters of peripheral blood were obtained from the median cubital vein using a 22-gauge needle and injected into four 9-mL sterile extraction tubes containing 3.8% trisodium citrate. Using the PRGF-Endoret system^®^ (BTI Biotechnology Institute, Bluebell, PA, USA), the blood was centrifuged once at 2100 rpm for 8 min at room temperature. A line was drawn 5 mm above the buffy-coat layer, and the supernate above this line was divided into two parts: the upper part was defined as platelet-poor plasma (PPP), and the lower part was defined as LP-PRP. After the PPP was removed, a total of 8 mL of LP-PRP was extracted; of this, 6 mL was injected into the knee joint, 1.5 mL was stored, and the remaining 0.5 mL was used for hematological analysis. The patient was placed in a supine position with the knee flexed at 20°. After confirming the presence of joint fluid by ultrasound, a puncture was performed if joint fluid was present, and an expert physician injected LP-PRP without using ultrasound. Six milliliters of prepared LP-PRP were injected into the suprapatellar pouch using a 21-gauge needle under sterile conditions. After injection, no restrictions were imposed on daily activities.

### 2.3. Hematological Analysis

The white blood cell (WBC) and platelet counts in whole blood (WB) samples and the LP-PRP were determined using an automated cell count analyzer (Sysmex KX-21 N, Kobe, Japan). The leukocyte contamination rates (number of LP-PRP WBCs/number of WB-WBCs) and the platelet concentration ratio (number of LP-PRP platelets/number of WB platelets) were also calculated.

### 2.4. Clinical Evaluation

Clinical outcomes were evaluated pre-injection and during the first post-injection outpatient visit. The Japanese Knee Osteoarthritis Measure (JKOM), which is a validated measure for Japanese patients with KOA [20], was used. The JKOM consists of five categories: category I, visual analog scale (VAS) (0–100); category II, pain and stiffness (pain; 0–32); category III, activities of daily living (ADL) (0–40); category IV, participation in social activities (social activity; 0–20); and category V, general health conditions (general health; 0–8). The total score amounts to 100 points (categories II–V). A higher JKOM score indicates a worse knee condition. A previous study has shown greater reliability and validity of the JKOM for clinical outcomes compared to other knee-related measures such as the Western Ontario and McMaster Universities Arthritis Index [22].

The OMERACT-OARSI [21] were modified to define responders and non-responders after LP-PRP injection. According to the OMERACT-OARSI criteria, responders were defined by “improvement in pain or function of at least 50% and absolute change of at least 20 points.” Moreover, responders were also defined by fulfillment of two of the following three criteria: “improvement in pain of at least 20% and absolute change of at least 10 points,” “improvement in function of at least 20% and absolute change of at least 10 points,” or “global improvement of at least 20% with an absolute change of at least 10 points.” Because JKOM is not included in the global score, in this study, those who fulfilled the “improvement in pain or function of at least 50% and absolute change of at least 20 points” criterion or both the “improvement in pain of at least 20% and absolute change of at least 10 points” and the “improvement in function of at least 20% and absolute change of at least 10 points” criteria were defined as responders. JKOM I (VAS) was used to evaluate “pain” and JKOM III (ADL), converted to a 100-point scale, was used to evaluate “function.”

### 2.5. Radiological Evaluation

The radiological severity of KOA was evaluated using the Kellgren/Lawrence (K/L) grade [23]. The hip–knee–ankle (HKA) angle was defined as the axis between the femoral mechanical axis and tibial mechanical axis. An HKA angle in varus alignment was marked positively. The joint convergence angle (JLCA) was measured as the angle between the line connecting the distal femur and the proximal tibial articular surfaces on anteroposterior radiographs in the standing and supine positions; these were defined as “stand JLCA” and “supine JLCA” respectively. If the apex of the JLCA was medial, it was recorded as positive (+) and denoted as varus. If it was lateral, it was recorded as negative (−) and denoted as valgus. The difference between stand JLCA and supine JLCA was defined as “ΔJLCA.”

### 2.6. Statistical Analyses

Continuous variables were presented as means ± standard deviations. The Shapiro–Wilk test was used to examine the normality of the distribution. The paired *t*-test was performed to compare normally distributed variables, and the Wilcoxon signed-rank test was performed to compare non-normally distributed variables between pre- and post- LP-PRP injection. The Student’s *t*-test was performed to compare normally distributed variables, the Mann–Whitney test was performed to compare non-normally distributed variables, and the Pearson’s chi-square test was performed to compare qualitative variables between responders and non-responders. The Wilcoxon signed-rank test was used to compare the pre- and post-injection results for each group. The Fisher’s exact test was used to compare qualitative variables. To investigate the patient factors that contributed to their response, a univariate regression analysis was performed initially; variables with a two-tailed *p*-value < 0.1 were used in the multivariable logistic regression analysis to calculate the standardized regression coefficients (B) odds ratios (OR) and 95% confidence intervals (CI). All statistical analyses were performed using the SPSS software (version 26.0; IBM Corp., Armonk, NY, USA). Statistical significance was set at *p* < 0.05.

## 3. Results

### 3.1. Demographic Data

Overall, 124 patients with a mean age of 70.3 ± 8.6 years were followed for an average of 3.3 ± 2.7 weeks. The demographic, biological, and radiological characteristics of the patients are summarized in Table 1. The obtained LP-PRP code was 24-00-00 according to the coding system [24].

### 3.2. Clinical Evaluation

All clinical outcomes, including the JKOM I–V scores, significantly improved after the LP-PRP injections (Table 2). The proportion of responders, defined above, was 58.1% (72 patients).

### 3.3. Comparison of Responder and Non-Responder Characteristics

The demographic, biological, and radiological characteristics of the responders and non-responders are summarized in Table 3.

### 3.4. Regression Analysis for Predicting Factors Associated with Responders

The univariate analysis revealed that the male sex (OR, 0.37; 95% CI, 0.16–0.86; *p* = 0.021), baseline JKOM I (VAS) (OR, 1.02; 95% CI, 1.01–1.04; *p* = 0.004), baseline JKOM II (pain) (OR, 1.08; 95% CI, 0.1.01–1.16; *p* = 0.032), platelet concentration ratio (OR, 3.26; 95% CI; 0.1.01–10.5; *p* = 0.032), and JLCA supine (OR, 0.83; 95% CI, 0.71–0.96; *p* = 0.048) were significantly associated with responders. The multivariate analysis revealed that JKOM I (VAS) scores at baseline (OR, 1.02; 95% CI, 1.01–1.04; *p* = 0.004), WBC count in whole blood (OR, 1.60; 95% CI, 0.1.14–223; *p* = 0.006), and platelet concentration ratio of the LP-PRP (OR, 5.27; 95% CI, 0.1.16–23.9; *p* = 0.031) were significantly associated with responders (Table 4).

## 4. Discussion

The important finding of this study was that a single injection of LP-PRP was effective in the short term for approximately 60% of the patients with KOA with K/L grades of 2–4.

Previous studies have shown varying responder rates to LP-PRP at 6 months, with one study reporting a rate of 52.8% [17] and another of 83% [25]. These results are not generally comparable, because the clinical scores of patients prior to LP-PRP injection were worse in studies with higher responder rates. In addition, although the current study only involved a single injection, previous studies have included three injections, with some studies showing that multiple injections were more effective than a single injection [26,27]. This should also be taken into consideration. In this study, the responder rate was approximately 60% for the very short term and was not inferior to the results at 6 months. Future research needs to focus on pathologies that are effective in the very short term and those that are effective in the medium term.

In this study, a multivariate logistic analysis was performed to identify factors contributing to LP-PRP efficacy, and higher JKOM I (VAS) scores at baseline, WB-WBC counts, and platelet concentration ratios of the LP-PRP were identified as contributing factors.

According to previous literature, the VAS score of a patient with KOA is not associated with the radiographic severity, but with the degree of synovitis [28]. This suggests that the pain in KOA is due to intra-articular inflammation. Furthermore, the main mechanism of pain relief by PRP is thought to be its anti-inflammatory effect secondary to NF-κB activity inhibition [29]. In one study, compared to controls, PRP prepared by different methods significantly improved synovitis on magnetic resonance imaging (MRI) [30] and reduced inflammatory cytokines, such as IL-1β and TNF-α, in the synovial fluid [31]. MRI is not the best option for evaluating synovitis, because it can only measure surrogates for inflammation, such as synovial thickening and effusion levels. In addition to evaluating synovitis with MRI, investigating pro-inflammatory cytokines in synovial fluid may provide new insights.

Regarding serum WBC, in a study comparing an OA group with an age- and sex-matched control group, the serum WBC count was found to be significantly higher in the OA group, suggesting that patients with OA are in a state of systemic inflammation [32]. Although there are no reports on the association between serum WBC and the clinical scores, IL-6 (a serum inflammatory marker) has been reported to be associated with the VAS score [33]. A detailed study on serum WBC and serum biomarkers suggestive of systemic inflammation as well as on the local synovial fluid findings in the knee may provide new insights into the pathogenesis of KOA [34].

The platelet concentration ratio is one of the indices used in the classification of PRP [35]. Although it has been reported that the platelet concentration ratio varies depending on the preparation method, the optimal platelet concentration ratio has not been clarified. The platelet concentration ratio of LP-PRP is approximately two-fold, and one characteristic of LP-PRP is that it can be prepared by manual aspiration. Although it is theoretically possible to increase the platelet concentration ratio, it should be noted that the leukocyte contamination rate will also increase with it [36]. One interesting finding was that the platelet concentration ratio in each individual was different even when LP-PRP was prepared using the same kit, which contributed to the response. The platelet concentration of LP-PRP correlated with the platelet-derived growth factor (PDGF)-BB [36,37], and PDGF has been reported to correlate with the clinical scores in PRP using different methods [38,39]. Further investigation into factors that determine the platelet concentration ratio and the growth factors in LP-PRP will help us to better understand the effects of LP-PRP therapy on KOA.

There is no consensus on the association between the radiological severity of KOA and the efficacy of PRP. Some reports have indicated that efficacy decreases with increasing severity of KOA [40,41,42], while others have reported that the degree of cartilage damage is not related to the efficacy of PRP [43]. In this study, significant differences in the HKA and supine-JLCA, but not in the ΔJLCA (which assesses knee instability), were noted between the responders and non-responders. This means that PRP has little effect if the varus alignment is severe, but knee joint instability is not related to the effect of LP-PRP. The pathology of KOA contains both mechanical and biological aspects, and LP-PRP may have an effect on the biological aspects.

There are several limitations to this study. First, this was a retrospective study that lacked a control group. Second, although the inclusion and exclusion criteria were defined, heterogeneous populations may have been assessed. Third, the effect of LP-PRP was investigated only for a very short interval. Clinical outcomes may improve over time [44], and longer-term studies are warranted to understand the effects of LP-PRP more thoroughly. Finally, growth factors in LP-PRP were not measured and detailed MRI evaluations were not performed.

In conclusion, LP-PRP improved the clinical scores in the short term. The patient characteristics before injection and the concentration ratio of LP-PRP may be predictors of LP-PRP efficacy. Further insights can be obtained by a detailed examination of the pathogenesis of KOA using joint fluid and MRI and of the growth factors within LP-PRP.

## Figures and Tables

**Table 1:** Patient demographics.

Variable	
Sex (male/female)	38/86
Age (years)	70.3 ± 8.6 (48–90)
Follow-up duration (weeks)	3.3 ± 2.7 (1–22)
K/L grade (2/3/4)	13/54/57
WB-WBC (×10^3^/μL)	6.2 ± 1.5
WB-Plt (×10^3^/μL)	238.2 ± 61.4
LP-PRP WBC (×10^3^/μL)	0.002 ± 0.01
LP-PRP Plt (×10^3^/μL)	453.4 ± 132.6
Leukocyte contamination rate	0.23 ± 1.4
Plt concentration ratio	1.92 ± 0.42

K/L: Kellgren/Lawrence; WB: whole blood; WBC: white blood cell; Plt: platelet; LP-PRP: leukocyte-poor platelet-rich plasma.

**Table 2:** Clinical outcomes after treatment with plasma rich in growth factors.

Parameter	Baseline	Follow-Up	*p*-Value
JKOM I (VAS)	50.7 ± 24.0	29.0 ± 23.6	<0.001
JKOM II (pain)	13.9 ± 5.2	8.6 ± 5.0	<0.001
JKOM III (ADL)	12.7 ± 7.2	8.5 ± 6.7	<0.001
JKOM IV (social activity)	6.5 ± 4.9	5.0 ± 4.6	<0.001
JKOM V (health condition)	3.2 ± 1.6	2.3 ± 1.6	<0.001
JKOM total	36.4 ± 15.9	24.4 ± 15.5	<0.001

JKOM: Japanese Knee Osteoarthritis Measure; VAS: visual analog scale; ADL: activities of daily living.

**Table 3:** Baseline characteristics of the responders and non-responders.

Parameter	Responders (*n* = 72)	Non-Responders (*n* = 52)	*p*-Value
Sex (male/female)	28/44	10/42	0.02
Age (years)	71.7 ± 8.7	70.5 ± 7.7	0.97
Baseline JKOM			
JKOM I (VAS)	56.3 ± 20.5	43.1 ± 25.4	0.004
JKOM II (pain)	14.6 ± 5.0	12.7 ± 5.2	0.04
JKOM III (ADL)	13.5 ± 7.2	11.7 ± 7.4	0.11
JKOM IV (social activity)	6.9 ± 4.7	6.4 ± 5.5	0.40
JKOM V (health condition)	2.9 ± 1.4	3.4 ± 1.7	0.52
JKOM total	37.9 ± 15.8	34.7 ± 16.5	0.10
WB-WBC (×103/μL)	6.7 ± 1.8	5.9 ± 1.3	0.09
WB-Plt (×103/μL)	241.1 ± 67.8	235.3 ± 54.2	0.78
LP-PRP WBC (×103/μL)	0.008 ± 0.04	0.02 ± 0.12	0.41
LP-PRP Plt (×103/μL)	472.2 ± 140.2	431.2 ± 111.0	0.15
Leukocyte contamination rate	0.08 ± 0.43	0.36 ± 2.0	0.41
Plt concentration ratio	2.0 ± 0.52	1.8 ± 0.24	0.02
K/L grade (2/3/4)	9/35/28	4/19/29	0.17
HKA (degree)	8.5 ± 5.5	10.1 ± 6.21	0.05
JLCA supine (degree)	2.6 ± 2.1	3.8 ± 2.9	0.005
JLCA stand (degree)	4.9 ± 3.0	5.6 ± 3.7	0.127
ΔJLCA (degree)	2.2 ± 1.7	1.9 ± 1.6	0.37

JKOM: Japanese Knee Osteoarthritis Measure; VAS: visual analog scale; ADL: activities of daily living; WB: whole blood; WBC: white blood cell; Plt: platelet; LP-PRP: leukocyte-poor platelet-rich plasma; K/L: Kellgren/Lawrence; HKA: hip–knee–ankle; JLCA: joint convergence angle; ΔJLCA: stand JLCA−supine JLCA.

**Table 4:** Regression analysis.

Variable	Univariate Model				Multivariate Model			
	B	OR	95% CI	*p*	B	OR	95% CI	*p*
Sex (male/female)	−0.98	0.37	0.16–0.86	0.02	−0.44	0.64	0.24–01.76	0.39
Age (years)	−0.01	0.99	0.96–1.04	0.84				
Baseline JKOM								
JKOM I (VAS)	0.02	1.02	1.01–1.04	0.004	0.04	1.04	1.01–1.06	0.006
JKOM II (pain)	0.08	1.08	1.01–1.16	0.03	−0.01	0.99	0.88–1.12	0.92
JKOM III (ADL)	0.04	1.04	0.98–1.09	0.19				
JKOM IV (social activity)	0.01	1.01	0.94–1.09	0.79				
JKOM V (health condition)	−0.11	0.90	0.72–1.13	0.37				
JKOM total	0.02	1.02	0.99–1.04	0.19				
WB-WBC (×10^3^/μL)	0.24	1.27	0.99–1.64	0.06	0.47	1.60	1.14–2.23	0.006
WB-Plt (×10^3^/μL)	0.00	1.00	0.99–1.01	0.65				
LP-PRP WBC (×10^3^/μL)	−1.41	0.24	0.01–14.5	0.50				
LP-PRP Plt (×10^3^/μL)	0.01	1.00	0.99–1.01	0.12				
Leukocyte contamination rate	−12.8	0.00	0.00–965	0.42				
Plt concentration ratio	1.18	3.26	1.01–10.5	0.04	1.66	5.27	1.16–23.9	0.03
KL grade	−0.51	0.60	0.34–1.05	0.07	−0.66	0.51	0.24–1.13	0.10
HKA (degree)	−0.05	0.95	0.89–1.01	0.12				
JLCA supine (degree)	−0.01	0.83	0.71–0.96	0.01	−0.16	0.86	0.70–1.05	0.06
JLCA stand (degree)	−0.07	0.93	0.84–1.03	0.22				
ΔJLCA (degree)	0.15	1.16	0.93–1.46	0.19				

OR, odds ratio; CI, confidence interval; JKOM: Japanese Knee Osteoarthritis Measure; VAS: visual analog scale; ADL: activities of daily living; WB: whole blood; WBC: white blood cell; Plt: platelet; LP-PRP: leukocyte-poor platelet-rich plasma; K/L: Kellgren/Lawrence; HKA: hip–knee–ankle; JLCA: joint convergence angle; ΔJLCA: stand JLCA−supine JLCA.

## Data Availability

The data presented in this study are available on request from the corresponding author.
